# TreeProfiler: large-scale metadata profiling along gene and species trees

**DOI:** 10.1093/molbev/msag028

**Published:** 2026-02-02

**Authors:** Ziqi Deng, Claudia Sanchis-López, Ana Hernández-Plaza, Adrián A Davín, Jaime Huerta-Cepas

**Affiliations:** Centro de Biotecnología y Genómica de Plantas, Universidad Politécnica de Madrid (UPM) and Instituto Nacional de Investigación y Tecnología Agraria y Alimentaria (INIA-CSIC), Madrid 28223, Spain; Centro de Biotecnología y Genómica de Plantas, Universidad Politécnica de Madrid (UPM) and Instituto Nacional de Investigación y Tecnología Agraria y Alimentaria (INIA-CSIC), Madrid 28223, Spain; Centro de Biotecnología y Genómica de Plantas, Universidad Politécnica de Madrid (UPM) and Instituto Nacional de Investigación y Tecnología Agraria y Alimentaria (INIA-CSIC), Madrid 28223, Spain; Department of Biology, Institute of Microbiology and Swiss Institute of Bioinformatics, ETH Zürich, Zurich, Switzerland; Centro de Biotecnología y Genómica de Plantas, Universidad Politécnica de Madrid (UPM) and Instituto Nacional de Investigación y Tecnología Agraria y Alimentaria (INIA-CSIC), Madrid 28223, Spain

**Keywords:** phylogenetic profiling, tree visualization, tree annotation, phylogenetics

## Abstract

Profiling biological traits along gene or species tree topologies is a well-established approach in comparative genomics, widely employed to infer gene function from co-evolutionary patterns (phylogenetic profiling), reconstruct ancestral states, and uncover ecological associations. However, existing profiling tools are typically tailored to specific use cases, have limited scalability for large datasets, and lack robust methods to aggregate or summarize traits at internal tree nodes. Here, we present TreeProfiler, a tool for automated annotation and interactive exploration of hundreds of features along large gene and species trees, with seamless summarization of mapped traits at internal nodes. TreeProfiler supports the profiling of custom continuous and discrete traits, as well as ancestral character reconstruction and phylogenetic signal tests. It also integrates commonly used genomic features, including multiple sequence alignments, protein domain architectures, and functional annotations. We demonstrate TreeProfiler's utility beyond traditional phylogenetic profiling, as well as its ability to efficiently handle massive datasets, by analyzing the functional diversification of the methyl-accepting chemotaxis protein family comprising over 400,000 genomic and metagenomic sequences and by profiling the relative abundance of 124,295 bacterial and archaeal species across 51 biomes. TreeProfiler is open-source and freely available at https://github.com/compgenomicslab/TreeProfiler.

## Introduction

Phylogenetic profiling was originally developed to compare the co-occurrence of genes across species to infer functional associations between proteins (Pellegrini et al. 1999). Since then, it has evolved into a tree-based comparative genomics approach used to analyze the distribution of one or more traits across species or genes while accounting for their evolutionary relationships. Today, phylogenetic profiling is a versatile exploration tool with a wide range of applications (Moi and Dessimoz 2023), from inferring protein interactions and identifying synapomorphic traits (Peng et al. 2023) to detecting horizontal transfer (Ravenhall et al. 2015) and convergent evolution events (Rey et al. 2018). In microbial ecology, phylogenetic profiling has also become useful for studying functional and ecological patterns of genes and species across environmental samples (Asnicar et al. 2015).

Although this broad utility has led to the development of numerous bioinformatics tools, phylogenetic tree profiling remains challenging when applied to the large and complex data generated by current genomic studies. First, annotating phylogenetic trees often requires ad hoc pipelines and is typically restricted to small and medium datasets. In addition, most phylogenetic profiling tools tend to focus on traditional use cases, such as profiling orthologous groups (Hernández-Plaza et al. 2023; Rossier et al. 2024; Szklarczyk et al. 2025; Tran and Ebersberger 2025), protein function (Fang et al. 2021), or domain architectures (Cromar et al. 2016), offering limited options to profile custom metadata. Tree visualization software, including programming libraries such as ETE Toolkit and ggtree (Huerta-Cepas et al. 2016; Yu et al. 2017), standalone applications (Asnicar et al. 2015; Cantrell et al. 2021) and online platforms like tvBOT or iTOL (Xie et al. 2023; Letunic and Bork 2024), are therefore the default option for exploring large and customly annotated phylogenetic trees. However, visual representation and interactive exploration of very large annotated phylogenies is a major challenge, as trees and metadata are usually too large to render and iteratively navigate. Moreover, when large trees are collapsed, most visualization software still displays individual leaf annotations overimposed at the collapsed nodes, which can create misleading impressions of data enrichment. For instance, randomly distributed trait values may appear as uniformly present across the whole tree simply due to overlapping graphical elements rather than averaging true biological signal under each collapsed node (Figure S1).

To address these limitations, we have developed TreeProfiler, a bioinformatics tool designed to automate the annotation of very large phylogenetic trees with custom features, facilitating their interactive visualization. TreeProfiler supports a wide range of metadata to be annotated and propagated across internal nodes of a phylogenetic tree, representing either discrete or continuous traits. Furthermore, it can easily incorporate functional annotations, taxonomic data, multiple sequence alignments, and protein domain architectures into the profiling options.

Finally, TreeProfiler provides seamless integration with methods for ancestral character reconstruction of discrete characters (Ishikawa et al. 2019; Revell 2024), phylogenetic signal tests (Ribeiro et al. 2023), and estimation of lineage-specific traits (Mendler et al. 2019). Interactive exploration of phylogenetic profiles is powered by ETE Toolkit v4.0, enabling efficient and interactive exploration of trees with hundreds of thousands of nodes.

## Implementation and main features

TreeProfiler is implemented as a command-line tool for annotating and visualizing phylogenetic trees with metadata. It provides two subcommands: *treeprofiler-annotate* for computing profiles and annotating metadata onto trees and *treeprofiler-plot* for interactive visualization and exploration of profiled data.

### Computing phylogenetic profiles

TreeProfiler annotates phylogenetic trees with user-provided metadata and propagates this information from leaf to root. The *treeprofiler-annotate* command automatically detects the most appropriate data type for each trait and summarizes values at internal nodes accordingly, using descriptive statistics for continuous traits and frequency counts for discrete ones. For example, continuous traits can be summarized using standard descriptive statistics (mean, median, maximum, minimum, and standard deviation), whereas discrete traits such as binary presence/absence variables or categorical labels like GO terms or KEGG pathways are summarized by counting the frequency of each term or pathway across descendant leaves. Traits with multiple categorical values, such as genes annotated with several KEGG pathways, are automatically converted into binary presence/absence profiles to facilitate visualization and downstream analysis.

TreeProfiler can also perform ancestral trait analysis on selected annotation tracks, including ancestral character reconstruction by maximum parsimony or maximum likelihood (using PastML; Ishikawa et al. 2019), phylogenetic signal tests using delta statistic (Ribeiro et al. 2023), and lineage specificity metrics for boolean traits. These methods can be used for one or more annotation tracks, resulting in the annotation of internal tree nodes with the corresponding values.

Special data types are supported to link common genomic information with tree topology, such as multiple sequence alignments, domain architectures, functional annotations, and taxonomic assignments. Thus, TreeProfiler can automatically interpret taxonomic annotations based on NCBI Taxonomy IDs (Sayers et al. 2019), GTDB (Parks et al. 2022), and mOTUs (Dmitrijeva et al. 2025), which are further used to infer full lineage tracks, species names, taxonomic rank, and last common ancestry along all tree nodes. Similarly, functional annotations and protein domain coordinates generated by eggNOG-mapper (Cantalapiedra et al. 2021), a widely used tool for functional annotation of genomes and metagenomes, are seamlessly integrated, allowing users to explore gene phylogenies alongside the distribution of, for example, Gene Ontology terms, KEGG annotations or Pfam protein domain architectures (Mistry et al. 2021). Additionally, multiple sequence alignments can be associated with their respective phylogenies, enabling automatic calculation of consensus sequences at internal nodes.

The output of *treeprofiler-annotate* is an annotated tree file with embedded metadata, which can be further used for programmatic analysis or custom visualization.

### Visualizing phylogenetic profiles and metadata

**Figure 1:**
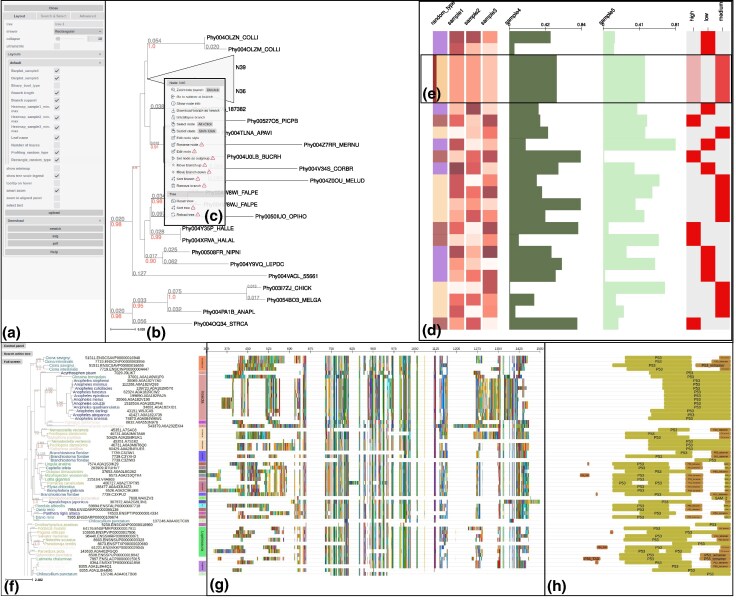
Overview of the *treeprofiler-plot* interface. a) Control panel for customizing visualization layouts, enabling annotation tracks, and managing search options. b) Phylogenetic tree viewer displaying support values (red) and branch lengths (gray). c) Node context menu with options for collapsing, pruning, rooting, and other per-node actions. d) Phylogenetic profiles of various data types visualized across the tree, including categorical traits (first column of color-coded rectangles), numeric traits displayed as heatmap gradients (columns 2 to 4) and bar plots (columns 5 to 6), as well as binary presence/absence profiles for categorical traits (final column). Column headers indicate the annotation track names. e) Summarized annotations for a collapsed clade: frequency distribution of categorical value is shown as stacked bar (column 1), averaged heatmap values (columns 2 to 4), averaged barplot values (columns 5 to 6) and presence/absence matrix gradients (positive-to-total ratio) at the last column. f) A phylogenetic tree annotated with taxonomic annotation and visualized with phylum-level classification using vertical color bands on corresponding clades. Leaf nodes are labeled with scientific names based on NCBI taxonomy. g) Multiple sequence alignment used to infer the tree. h) Most frequent protein domain architectures identified under each tree branch.

The *treeprofiler-plot* command allows users to interactively visualize and explore previously annotated trees, letting them decide and change the appropriate graphical layout used for each annotation track. The graphical user interface of *treeprofiler-plot* is based on the newest ETE Toolkit drawing engine (v4), which provides support for the interactive navigation of very large phylogenies (eg >100,000 tree nodes), as well as dynamic control of what annotation tracks are visible (Fig. 1a).

Continuous traits can be visualized as text labels, heatmaps, bar plots, or color gradients; while categorical traits can be shown as colored labels, symbols, or converted into heatmap-based presence/absence matrices (Fig. 1d). Importantly, when clades are collapsed and leaf nodes are not visible, TreeProfiler will display the annotations inferred for internal nodes, representing the statistical summary of all values collapsed under each node. For instance, instead of graphically overlapping the representation of multiple leaf values, the average, maximum, minimum, or frequency distribution of the grouped leaves will be shown (Fig. 1e and Figure S1). Special annotation tracks have their own dedicated layouts and graphical style configurations. Thus, taxonomic classifications, including common ancestry, are represented as vertical color bands aligned with their corresponding clades (Fig. 1f); multiple sequence alignments and consensus sequence representations are dynamically adjusted to the zoom level (Fig. 1g); and the most frequent protein domain architecture is shown for each branch (Fig. 1h).

Furthermore, *treeprofiler-plot* offers options for setting up custom node collapsing and highlighting rules based on tree node annotations. For example, large trees can automatically collapse at a specific taxonomic rank or at internal nodes that meet particular size or annotation value criteria.

Finally, to facilitate tree profile visualization of large data tables, the helper command *treeprofiler-desktop* is available, which will launch a web-based interface to guide the exploration of all annotation tracks available in a tree, as well as their possible visualization options and layouts offered by TreeProfiler.

### Use case 1: novel evolutionary and functional relationships of chemoreceptors (CRs) revealed by the MCPsignal domain

To demonstrate the capacity of TreeProfiler as a discovery tool for large-scale metagenomic analyses, we applied it to explore the functional landscape of one of the largest and most biodiverse bacterial protein families: the methyl-accepting chemotaxis proteins (MCPs). MCPs are multi-domain signal transduction proteins typically composed of an extracellular ligand-binding domain and a conserved cytoplasmic signaling domain (Alexander and Zhulin 2007; Ortega et al. 2017). They play a central role in microbial chemosensory processes, detecting environmental cues and transmitting these signals through the conserved MCP signaling domain, which interacts with CheA histidine kinases to regulate downstream responses. The ability of each MCP to interact with different CheA subtypes can determine distinct cellular behaviors (Alexander and Zhulin 2007).

However, the vast size and structural diversity of the MCP family make comprehensive phylogenetic and functional profiling particularly challenging, often exceeding the analytical and visualization capacities of conventional tree-viewing tools. A recent phylogenomic and ecological analysis (Sanchis López 2025) combined genomic and metagenomic data to produce an extensive phylogeny comprising over 400,000 MCP sequences, a dataset that is difficult to explore with existing visualization platforms.

**Figure 2:**
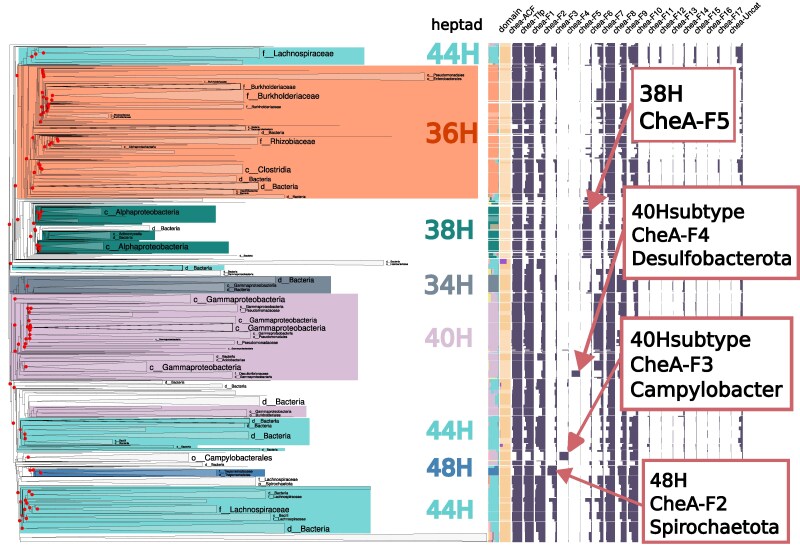
Phylogenetic profiling analysis of bacterial chemoreceptor (MCP) diversity. The tree was constructed from 428,179 unique MCP sequences and is annotated with two primary features. First, branch colors represent the dominant heptad structural class (applied where sequence uniformity is ≥90%). Second, the adjacent heatmap indicates the co-occurrence of CheA signaling subtypes genes (ACF, Tfp, F1–F17) within the same genomes, uncovering potential functional associations with specific MCP clades.

Here, we combined this large-scale phylogeny with functional and genomic metadata to investigate the evolutionary links between MCP structural variants and their associated CheA signaling systems. MCP signaling domains are characterized by a repeating, seven-amino-acid unit known as a heptad, which forms the coiled-coil interface for interaction with CheA. Different “heptad classes” (eg 38H, 48H) define major MCP structural subtypes. By mapping these data onto the MCPsignal tree and summarizing them hierarchically by dominant heptad composition, TreeProfiler's visualization revealed previously unrecognized global evolutionary correlations between MCP heptad classes and CheA subtypes, highlighting lineage-specific diversification and co-evolution within this protein family (Fig. 2).

Specifically, the visualization uncovered the CheA F5 system and its strong correlation with the 38H heptad class. Furthermore, it revealed a previously unreported association between the 48H subtype and F2 systems in *Treponema (Spirochaetota)*, consistent with earlier observations of the structural specialization of F2 arrays (Muok et al. 2020). Additional patterns identified through high-level profiling and internal node summarization included a *Campylobacter*-specific 40H-like subtype linked to F3 systems and a distinct 40H subgroup associated with F4 systems in *Desulfobacterota*. Together, these patterns reveal a complex mosaic of co-evolution and subfunctionalization among MCP structural classes and CheA systems, demonstrating how large-scale visualization with TreeProfiler can uncover evolutionary and functional relationships that remain inaccessible to conventional analyses.

### Use case 2: resolving the ecological mosaic of *Patescibacteria* across global biomes

**Figure 3:**
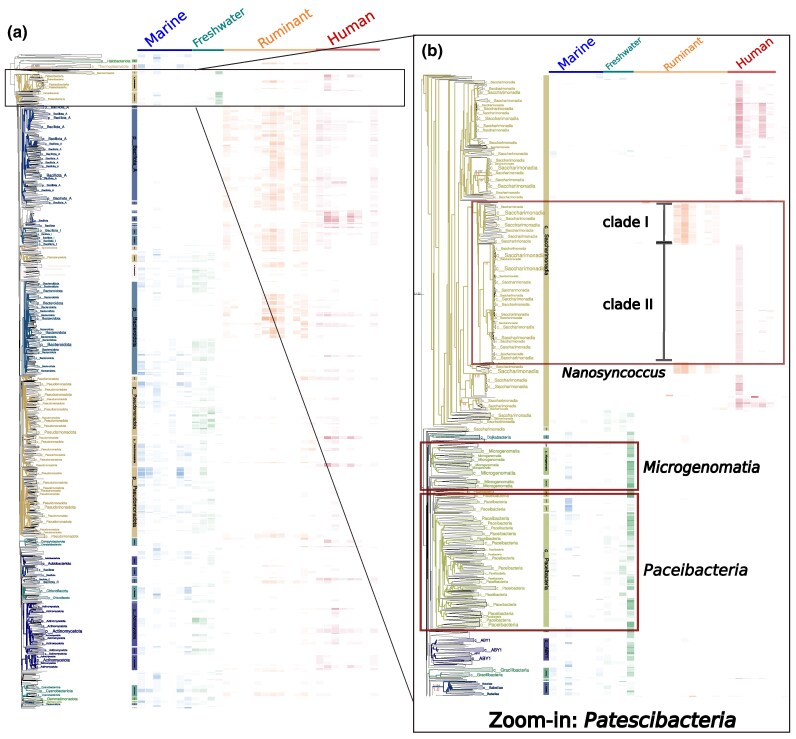
Hierarchical exploration of ecological patterns across the mOTUs reference tree. a) Global overview of the complete phylogeny colored by phylums with phylum-level LCAs marked by vertical color bands, annotated with relative abundance across selected biomes. *Patescibacteria* clades are outlined in black for next zoom in. b) Zoom-in view of *Patescibacteria* at the class level. Environmental classes (*Pacisbacteria*, *Microgenomatia*) contrast with the host-associated *Saccharimonadia*. The *Nanosyncoccus* genus is highlighted in red, revealing two major subclades with contrasting abundance patterns in human oral versus ruminant-associated samples.

To showcase TreeProfiler's ability to extract ecological and evolutionary insights from large metagenomic datasets, we applied it to the global prokaryotic phylogeny reconstructed from the mOTUs reference catalog (Dmitrijeva et al. 2025). This dataset includes 124,295 archaeal and bacterial species with abundance estimates across 112,121 metagenomic samples from 51 biomes, offering an unprecedented opportunity to visualize ecological distributions along the prokaryotic tree of life and identify lineages with distinct environmental patterns (Fig. 3a).

As an example, we examined the ecological distribution of lineages within the phylum *Patescibacteria* (candidate phyla radiation). Members of this phylum, characterized by small cell size, streamlined genomes, and episymbiotic lifestyles, are frequently detected in groundwater, freshwater, and animal-associated habitats (McLean et al. 2020; Hu et al. 2024; Srinivas et al. 2024). However, ecological differentiation among its internal lineages remains poorly understood.

TreeProfiler's interactive profiling revealed clear habitat partitioning among *Patescibacteria* classes (Fig. 3b). *Paceibacteria* and *Microgenomatia* were enriched in groundwater and freshwater environments, whereas *Saccharimonadia* showed a distinct host-associated pattern. At finer resolution, it uncovered a pronounced ecological split within the genus *Nanosyncoccus*: one clade (∼1,200 genomes) enriched in human oral samples, and another (∼370 genomes) predominant in mammalian (particularly ruminant) gut metagenomes. This bifurcation was consistent across biomes and abundance metrics, indicating lineage-specific ecological specialization. Both clades comprise largely uncultivated representatives, extending beyond the few cultivated oral *Saccharimonadia* species isolated from human oral and wastewater environments (He et al. 2024).

Previous genomic surveys noted habitat adaptation across *Saccharimonadia*, but not this within-genus, biome-specific diversification. By integrating abundance data from over 100,000 metagenomes with species-level phylogeny, TreeProfiler's dynamic zooming and node summarization effectively revealed ecological patterns obscured in massive datasets.

## Conclusions

TreeProfiler overcomes current limitations of existing tools for phylogenetic tree profiling by offering a flexible framework for annotating, summarizing, and interactively visualizing custom metadata across large phylogenetic trees. These capabilities enable the exploration of ancestral trait distributions, lineage-specific patterns, and clade-level summaries within complex evolutionary contexts. The entire workflow is streamlined into two command-line steps, eliminating the need for ad hoc pipelines and enabling the rapid analysis of large-scale datasets.

## Supplementary Material

msag028_Supplementary_Data

## Data Availability

Comprehensive documentation and usage examples are available at https://github.com/compgenomicslab/TreeProfiler, with code and figure reproduction instructions at https://github.com/dengzq1234/treeprofiler_paper.
